# Müllerian Cyst of the Vagina Masquerading as a Cystocele

**DOI:** 10.1155/2015/376834

**Published:** 2015-01-31

**Authors:** Emrah Töz, Muzaffer Sancı, Süheyla Cumurcu, Aykut Özcan

**Affiliations:** ^1^Department of Gynecology and Oncology, İzmir Tepecik Education and Research Hospital, 35330 İzmir, Turkey; ^2^Department of Pathology, İzmir Tepecik Education and Research Hospital, 35330 İzmir, Turkey

## Abstract

Müllerian cysts are usually small, ranging from 0.1 to 2 cm in diameter. Rarely, they may be enlarged and mistaken for other structures, such as a cystocele or urethral diverticulum. We report on a female with symptomatic vaginal wall prolapse, diagnosed as a vaginal Müllerian cyst, which was originally misdiagnosed as a cystocele. The mass was soft and could be compressed manually without difficulty. Perineal ultrasonography and cystoscopy revealed no relationship between the cyst and the lower urinary tract, suggesting independence of the lesion. We performed surgical treatment with complete excision of the mass via a vaginal approach under spinal anaesthesia. The pathology result confirmed a benign Müllerian cyst lined with mucinous and squamous epithelium. When evaluating an anterior vaginal cyst, assessment of the lesion via history taking and pelvic examination is important to confirm both lesion size and location. Perineal ultrasonography performed with an empty bladder is useful to differentiate such vaginal cysts and to define their communication, if any, with adjacent organs.

## 1. Introduction

The prevalence of vaginal cysts is unclear but it is estimated to be <1%. They typically present in patients in the third or fourth decade of life [1]. Their origin may be Müllerian (paramesonephric), Wolffian (mesonephric), squamous (traumatic), or urogenital. The cysts are usually small, ranging from 0.1 to 2 cm in diameter, although they may measure >4 cm. The differential diagnoses of a cyst in the lower female genital tract include Müllerian cyst, inclusion cyst, mesonephric cyst (Gartner's duct), Bartholin gland cyst, urethrocele, urethral diverticulum, Skene's duct cyst, pelvic organ prolapse, hematocolpos, and a myxomatous tumour [2]. Simple mesonephric (Gartner's) or paramesonephric (Müllerian) cysts may occur at especially high levels near the fornices. Gartner's duct cysts are less common than Müllerian cysts, comprising approximately 10% of benign vaginal cysts; such cysts are almost always located along the lateral wall of the vagina. Gartner's duct cysts can also be associated with abnormalities of the metanephric urinary system such as an ectopic ureter, unilateral renal agenesis, and renal hypoplasia. Imaging by ultrasound or magnetic resonance imaging (MRI) is essential to identify the exact location of a cyst, the borders thereof, and the extent of communication with the adjacent organs and the urinary tract.

Müllerian cysts are the most common type of vaginal cysts, accounting for up to 40% of cystic masses [3]. During replacement of Müllerian epithelium with squamous epithelium of the urogenital sinus, Müllerian epithelial tissue can persist anywhere in the vaginal wall. The cysts can thus be found almost anywhere within the vaginal walls. However, the most common location is along the anterolateral aspect of the vagina. The great majority of cysts are asymptomatic and require no treatment. Occasionally, a Müllerian cyst may become sufficiently large that symptoms warrant excision. Cysts derived from the Müllerian ducts may exhibit histological patterns corresponding to those of any of the tissues normally derived from this duct, principally endocervical, tubal, or endometrial. Müllerian cysts with an endocervical (mucinous) lining are the most common. These are usually small, although a few may become quite large, as in our case. We report a female with symptomatic vaginal wall prolapse diagnosed as a vaginal Müllerian cyst. We discuss this unusual case of a large, symptomatic Müllerian cyst, which was misdiagnosed as a cystocele.

## 2. Case Report

**Figure 1:**
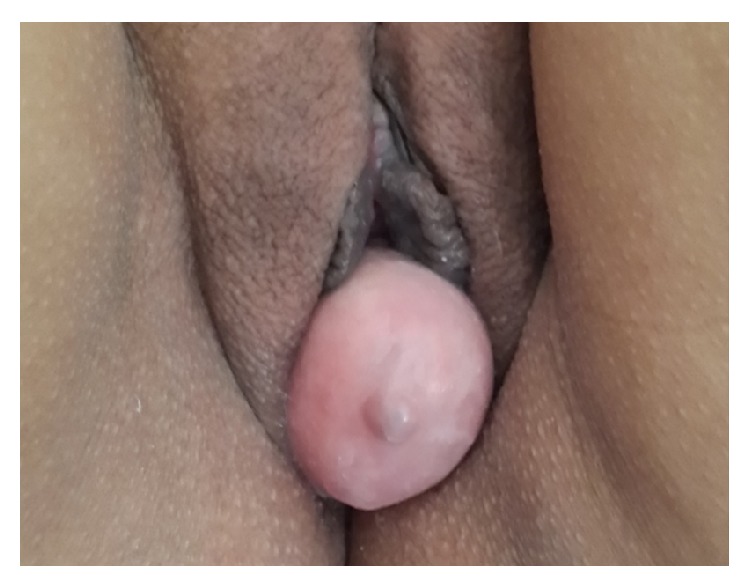
A pelvic examination revealed a mass of the anterior wall of the vagina measuring 8 cm in diameter.

A 42-year-old woman, para 2, originally diagnosed with a cystocele, was referred to our gynecological clinic because of a nontender mass protruding from her vagina. The mass had been evident for about 6 months, without any symptoms. Her medical history was unremarkable. A pelvic examination revealed a mass of the anterior wall of the vagina measuring 8 cm in diameter (Figure 1).

**Figure 2:**
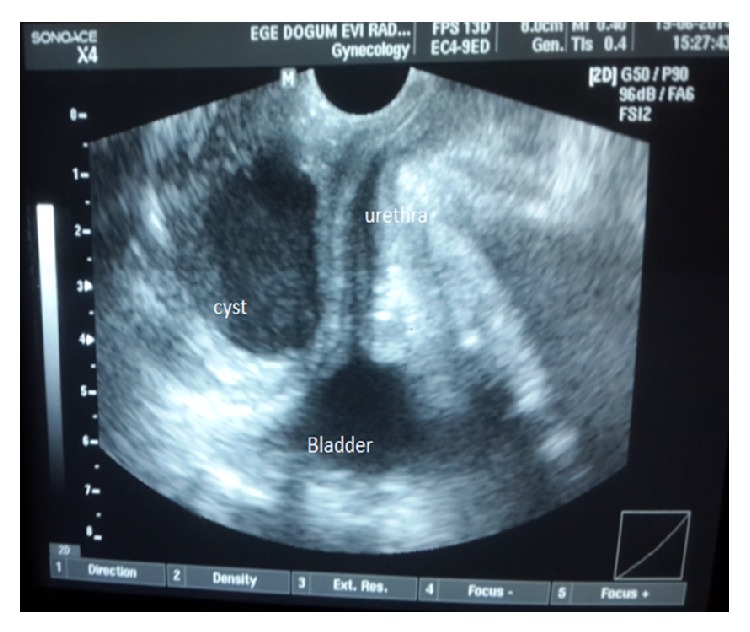
Perineal ultrasonography revealed no relationship between the cyst and the lower urinary tract.

The mass was soft and could be compressed manually without difficulty. Perineal ultrasonography and cystoscopy showed that no relationship existed between the cyst and the lower urinary tract, suggesting independence of the lesion (Figure 2). After the bladder was emptied, we evaluated the patient again, and the cyst persisted without any change in size.

**Figure 3:**
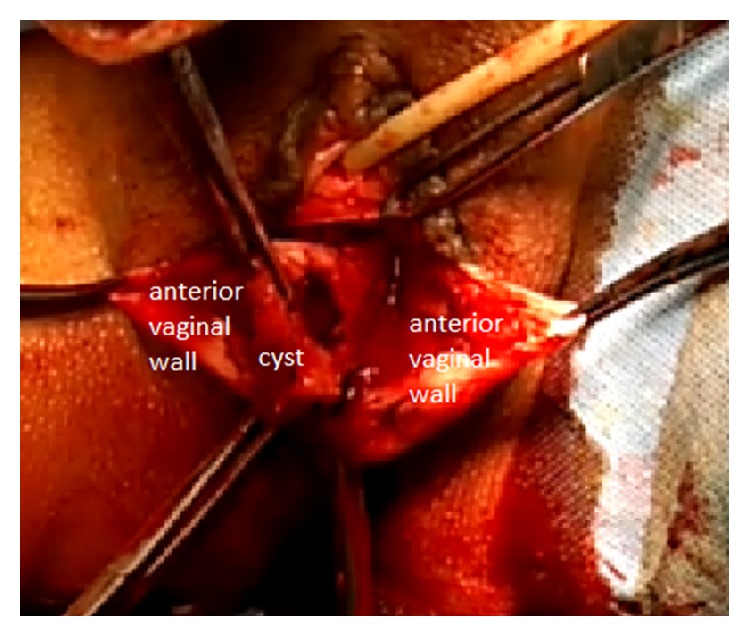
Surgical treatment with complete excision of the mass via a vaginal approach.

Abdominal and pelvic ultrasonography excluded any renal or associated internal genital pathology. We performed surgical treatment featuring complete excision of the mass via a vaginal approach, under spinal anaesthesia (Figure 3).

**Figure 4:**
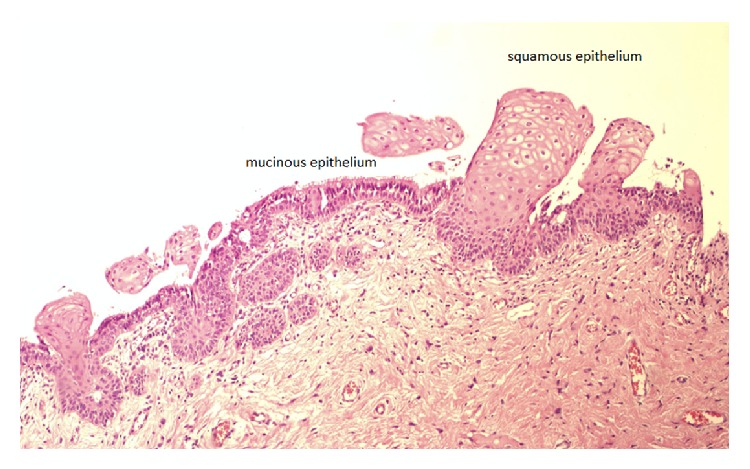
Cyst is lined by columnar mucin producing epithelium and squamous epithelium.

No complications were observed postoperatively, and at the 4-week follow-up visit, the patient was in good clinical condition. Pathology confirmed a benign Müllerian cyst lined with mucinous and squamous epithelium (Figure 4).

## 3. Discussion

Vaginal wall cysts tend to be embryological in nature and often asymptomatic. Müllerian cysts usually present as small, midline cystic masses with no symptoms and require no treatment. When cysts are symptomatic, patients may present with vaginal discomfort, vaginal pressure, vaginal mass, dyspareunia, vaginal bleeding, or urinary symptoms. Müllerian cysts are benign, and malignant transformation has been reported only once [4]. When the cysts enlarge, they may be mistaken for other structures, such as a cystocele or a urethral diverticulum. This case differs from the norm in that the cyst grew in size and became increasingly symptomatic. Imaging by ultrasound or MRI is essential for diagnosis.

If Müllerian cysts are asymptomatic, they are best left untreated. If they are symptomatic, before embarking on an operation to remove the cyst, it is important to obtain as much preoperative information about the cyst and the neighbouring structures as possible. Ultrasonography has the advantage that it is an inexpensive, real-time diagnostic procedure. However, MRI affords excellent visualisation of the vagina and surrounding tissue, offering high-contrast resolution and multiplanar capabilities [5]. In our case, diagnosis was performed only via ultrasound because the information yielded by ultrasound was adequate. We did not use MRI because of cost.

Vaginal cysts are treated via excision. The technique for all cysts is similar. The entire cyst wall must be removed to prevent recurrence. However, on occasion, cysts may extend cranially throughout the entire length of the vagina and (via the cervix) into the broad ligament. This renders it impossible to remove the entire cystic capsule. In such cases, if a portion of the cyst remains unresected, the interior of the capsule should be vaporised to diminish the chances of recurrence.

The differential diagnosis of Müllerian and Gartner's duct cysts requires histochemical evaluation of epithelial mucin production. In contrast to the epithelium of Müllerian cysts, the epithelium of Gartner's cysts is devoid of cytoplasmic mucicarmine and PAS-positive material [6].

In conclusion, this is an unusual case of an anterior vaginal cyst mimicking a cystocele, caused by a Müllerian cyst in a premenopausal female. When evaluating an anterior vaginal cyst, assessment of the lesion via history taking and pelvic examination is important to confirm lesion size and location. Perineal ultrasonography with an empty bladder is useful to differentiate the vaginal cysts and their communication, if any, with adjacent organs.
